# Blood microRNA expressions in patients with mild to moderate psoriasis and the relationship between microRNAs and psoriasis activity^⋆^^⋆⋆^

**DOI:** 10.1016/j.abd.2020.07.001

**Published:** 2020-08-05

**Authors:** Emine Tugba Alatas, Murat Kara, Gursoy Dogan, Aslı Akın Belli

**Affiliations:** a Department of Dermatology, School of Medicine, University of Mugla, Turkey; b Department of Medical Genetics, School of Medicine, University of Mugla, Turkey; c Erdem Hospital, İstanbul, Turkey

**Keywords:** Biomarkers, MicroRNAs, Pharmacological, Psoriasis

## Abstract

**Background:**

In recent studies, microRNAs (mi-RNAs) have been shown to play an important role in psoriasis pathogenesis. However, studies evaluating mi-RNAs in the blood of psoriasis patients including a large number of mi-RNA panels are scarce.

**Objective:**

The authors aimed to assess mi-RNA expressions in blood samples of psoriasis patients, as well as to evaluate the association between mi-RNA expression and psoriasis severity.

**Methods:**

This was a case-control study on 52 patients with psoriasis vulgaris and 54 controls. Patients’ medical history, psoriasis area and severity index (PASI) scores, and dermatology life quality index (DLQI) scores were recorded. The 42 disease-related mi-RNA primers were assessed by real-time PCR.

**Results:**

In the patient group, 13.4% presented nail involvement and 8.2% had psoriatic arthritis. The mean PASI and DLQI scores were 7.90 ± 8.83 and 8.13 ± 5.50, respectively. Among 42 mi-RNA primers; hsa-miR-155-5p, hsa-miR-369-3p, hsa-miR-193b-3p, hsa-miR-498, hsa-miR-1266-5p, hsa-let-7d-5p, hsa-miR-205-5p, hsa-let-7c-5p, hsa-miR-30b-3p, and hsa-miR-515-3p expressions were significantly up-regulated, whereas hsa-miR-21-5p, hsa-miR-142-3p, hsa-miR-424-5p, hsa-miR-223-3p, hsa-miR-26a-5p, hsa-miR-106b-5p, hsa-miR-126-5p, hsa-miR-181a-5p, hsa-miR-222-3p, hsa-miR-22-3p, hsa-miR-24-3p, hsa-miR-17-3p, hsa-miR-30b-5p, hsa-miR-130a-3p, hsa-miR-30e-5p, and hsa-miR-16-5p were significantly down-regulated in psoriasis patients when compared with the control group (*p* < 0.05).

**Study limitations:**

As the study included patients with mild to moderate psoriasis who mostly only received topical treatments, changes in miRNA before and after systemic treatments were not assessed.

**Conclusion:**

The detection of 24 mi-RNA expressions up- or down-regulated in psoriasis patients, even in those with milder disease, further supports the role of mi-RNAs in the psoriasis pathogenesis. Future studies should clarify whether mi-RNAs can be used as a marker for psoriasis prognosis or as a therapeutic agent in the treatment of psoriasis.

## Introduction

Psoriasis is a chronic, hyperproliferative, inflammatory skin disease characterized by erythematous squamous plaques, with a genetic etiology.^1^, ^2^, ^3^ While psoriasis is observed in 1–3% of the general population, psoriasis patients constitute 6–8% of the patients admitted to dermatology clinics.^4^ Although the pathogenesis of psoriasis cannot be fully explained, it is considered to be a polygenic disease that interacts with genetic and environmental factors. The primary antigen-resolving HLA-Cw6 gene, which is located in PSORS1 and encodes a major histocompatibility complex I allele, has been significantly associated with psoriasis. Changes in PSORS1 and PSORS2 genes have been observed in the development of psoriasis, by affecting immune cells and keratinocytes. Genetic factors have an impact on clinical symptoms, age at disease onset, type, and severity of psoriasis.^5^, ^6^

MicroRNAs (mi-RNAs) are members of the small RNA family ranging in the length of 19–25 nucleotides.^7^ Over 2500 mi-RNAs that play a key role in the regulation of basic biological processes have been identified to date.^8^ Mi-RNAs regulate gene expression during transcription and may act as a tumor suppressor gene and/or oncogene in diseases or specific tissues.^9^ Recent studies have shown that mi-RNAs are involved in the pathogenesis of psoriasis.^10^, ^11^, ^12^, ^13^ After the first study demonstrating the association between mi-RNAs and psoriasis, over 250 mi-RNAs have been reported as aberrantly expressed in psoriasis.^14^ Diverse mi-RNA profiles have been studied in psoriatic skin, psoriatic patients’ blood, and hair samples. Genetic polymorphisms of specific mi-RNAs, such as miR-146a, have been associated with psoriasis pathogenesis.^15^, ^16^ However, in most of the available studies, mi-RNA expressions have been demonstrated in psoriatic skin lesions with relatively small mi-RNA panels.

The authors aimed to identify and characterize mi-RNA expressions in blood samples of psoriasis patients with a large mi-RNA panel and to investigate the relationship between mi-RNA expression and severity of psoriasis through Psoriasis Area and Severity Index (PASI) and Dermatology Life Quality Index (DLQI) scores.

## Materials and methods

### Study design

This was a case-control study that included 52 psoriasis patients and 54 healthy controls recruited at the Departments of Dermatology and Medical Genetics of Muğla Sıtkı Koçman University Medical School. Ethics committee approval was obtained from the Clinical Research Ethics Committee of Muğla Sıtkı Koçman University. Patients and controls signed an informed consent form. The study included 52 patients with psoriasis vulgaris and 54 age- and gender-matched volunteers who were admitted to the dermatology outpatient clinic and met the inclusion/exclusion criteria. Inclusion criteria for the study were those who agreed to participate in the study and age > 18 years. Exclusion criteria for the study were as follows: age < 18 years, patients with psoriasis other than plaque-type (pustular psoriasis and erythrodermic psoriasis, among others.), patients with any systemic diseases (such as coronary artery disease, liver failure, renal failure, and malignancy), and patients who were pregnant or breastfeeding.

The medical history of the patients (age, disease duration, presence of psoriatic arthritis and nail involvement, and current treatments) were recorded in patient forms. Disease severity was calculated using the PASI scoring method. In addition, the DLQI scores of the patients were recorded.

Of the patients, 2–3 cc of blood was collected in EDTA tubes and sent to the Medical Genetics Laboratory. Patients’ RNAs were stored at −80 °C until the study was performed. Using the miRNeasy Mini Kit (QIAGEN Sample & Assay Technologies – Hilden, Germany), mi-RNA was isolated in accordance with the manufacturer's recommended protocol. The miScript II RT Kit (Qiagen) was used to convert the obtained RNAs into a computational DNA (cDNA). Levels of mi-RNA expressions were determined by the Biomark Real-Time PCR (Fluidigm) device.

Forty-two mi-RNA primers thought to be related to psoriasis (hsa-miR-155-5p, hsa-miR-31-5p, hsa-miR-99a-5p, hsa-miR-128-3p, hsa-miR-146a-5p, hsa-miR-210-3p, hsa-miR-369-3p, hsa-miR-378a-3p, hsa-let-7i-5p, hsa-miR-193b-3p, hsa-miR-498, hsa-miR-551a, hsa-miR-1266-5p, hsa-let-7d-5p, hsa-miR-205-5p, hsa-let-7c-3p, hsa-let-7c-5p, hsa-miR-30b-3p, hsa-miR-130a 5p, hsa-miR-515-3p, hsa-miR-21-5p, hsa-miR-125b-5p, hsa-miR-142-3p, hsa-miR-424-5p, hsa-miR-223-3p, hsa-miR-425-3p, hsa-miR-26a-5p, hsa-miR-26b-5p, hsa-miR-106b-5p, hsa-miR-126-5p, hsa-miR-181a-5p, hsa-miR-221-3p, hsa-miR-222-3p, hsa-miR-22-3p, hsa-miR-24-3p, hsa-miR-17-3p, hsa-miR-17-5p, hsa-miR-30b-5p, and hsa-miR-130a-3p, hsa-miR-30e-5p, hsa-miR-16-5p, and hsa-miR-30a-5p) were analyzed.^10^, ^11^, ^12^, ^13^

In the patient group, the relationship between mi-RNA expressions and PASI scores, DLQI scores, and nail involvement was assessed.

### Data analysis

Statistical analysis of the study was performed with SPSS® for Windows, v. 15.0. The results were presented as mean ± standard deviation. The analysis was made on the website http://www.qiagen.com/us/shop/genes-and-pathways/data-analysis-center-overview-page/. RT-PCR results (Ct values) were calculated by the 2-ΔΔCT method. After these values were calculated, Student's *t*-test was used to make comparisons between groups (calculation of *p*-values); *p*-values <0.05 were considered to be statistically significant.

## Results

The study included 52 psoriasis patients (32 females, 20 males; age range: 18–8 years) and 54 controls (33 females, 21 males; age range: 18–69 years). The mean age was 46.3 ± 17.7 years and 43.5 ± 16.5 years in the patient and control groups, respectively (*p* > 0.05). While 7.6% of the patients were undergoing systemic therapy (3.8% cyclosporin and 3.8% acitretin), 92.4% were only being treated with topical treatments. In the patient group, 13.4% presented nail involvement and 8.2% had psoriatic arthritis. The mean PASI score was 7.90 ± 8.83 and the mean DLQI score was 8.13 ± 5.50 in the patient group (Table 1).

**Table 1:** Sociodemographic characteristics and biochemical parameters of the patient and control groups.

	Psoriasis (*n* = 52)	Control (*n* = 54)
Age (years)	46.30 ± 17.73	43.53 ± 16.52
Gender (female/male)	32/20	33/21
Topical treatment (%)	92.4	
Sistemic treatment (%)	7.6	
Acitretin (%)	3.8	
Cylosporin (%)	3.8	
Psoriatic arthritis (%)	8.2	
Nail involvement (%)	13.4	
PASI	7.90 ± 8.83	
DLQI	8.13 ± 5.50	

The up- and down-regulated mi-RNA expressions of patient and control groups are shown in Table 2, Table 3, respectively. The expressions of hsa-miR-155-5p, hsa-miR-369-3p, hsa-miR-193b-3p, hsa-miR-498, hsa-miR-1266-5p, hsa-let-7d-5p, hsa-miR-205-5p, hsa-let-7c-5p, hsa-miR-30b-3p, and hsa-miR-515-3p were found to be significantly up-regulated in the psoriasis group when compared with the control group (*p* < 0.05; Table 2).

**Table 2:** Up-regulated in the evaluation of miRNA expression between patient and control group.

miRNA	Control		Psoriasis vulgaris	
N=20	2˄(-Avg. (Delta(Ct))	2˄(-Avg. (Delta(Ct))	Fold Change	P-value
hsa-miR-155-5p	0,02983	0,033644	1,1279	0,03163

hsa-miR-31-5p	0,032959	0,035317	1,0715	0,8496

hsa-miR-99a-5p	0,053615	0,033017	1,0209	0,96027

hsa-miR-128-3p	0,02978	0,033138	1,1128	0,07311

hsa-miR-146a-5p	0,032379	0,033843	1,0452	0,60549

hsa-miR-210-3p	0,034191	0,034284	1,0027	0,7119

hsa-miR-369-3p	0,029113	0,033069	1,1359	0,01889

hsa-miR-378a-3p	0,043387	0,049672	1,1449	0,382

hsa-let-7i-5p	0,031176	0,033099	1,0617	0,63498

hsa-miR-193b-3p	0,029113	0,033017	1,1341	0,02016

hsa-miR-498	0,029113	0,033168	1,1393	0,01674

hsa-miR-551a	1,408725	1,461312	1,0373	0,24357

hsa-miR-1266-5p	0,029113	0,033017	1,1341	0,02016

hsa-let-7d-5p	0,029113	0,033017	1,1341	0,02016

hsa-miR-205-5p	0,02969	0,033017	1,1121	0,04596

hsa-let-7c-3p	0,030431	0,034069	1,1195	0,64995

hsa-let-7c-5p	0,029554	0,033017	1,1172	0,04084

hsa-miR-30b-3p	0,029113	0,033312	1,1442	0,0142

hsa-miR-130a-5p	0,029856	0,033552	1,1238	0,1268

hsa-miR-515-3p	0,029856	0,033552	1,1238	0,03631

˄, Up-regulation (red).

Fold-change and fold-regulation values greater than 2 are indicated in red.

**Table 3:** Down-regulated in the evaluation of miRNA expression between patient and control group.

miRNA	Control		Psoriasis vulgaris	
n=20	2˄(-Avg. (Delta(Ct))	2˄(-Avg. (Delta(Ct))	Fold Change	P-value
hsa-miR-21-5p	0,193773	0,045936	0,2371˅	0,00039

hsa-miR-125b-5p	0,053615	0,042915	0,8004	0,10844

hsa-miR-142-3p	0,107994	0,042771	0,3943 ˅	0,00214

hsa-miR-424-5p	0,147849	0,043429	0,2937 ˅	0,000103

hsa-miR-223-3p	1,911406	0,066345	0,0347 ˅	0,00067

hsa-miR-425-3p	0,709862	0,684317	0,964	0,20361

hsa-miR-26a-5p	0,060076	0,03822	0,6362	0,0107

hsa-miR-26b-5p	0,038971	0,033936	0,8708	0,08779

hsa-miR-106b-5p	0,059833	0,036577	0,6113	0,00391

hsa-miR-126-5p	0,048731	0,036668	0,7525	0,01229

hsa-miR-181a-5p	0,045429	0,035526	0,782	0,00952

hsa-miR-221-3p	0,039388	0,035623	0,9044	0,12515

hsa-miR-222-3p	0,097659	0,043537	0,4458˅	0,00102

hsa-miR-22-3p	0,092048	0,040819	0,4434˅	0,0005

hsa-miR-24-3p	0,128319	0,043497	0,339˅	0,00018

hsa-miR-17-3p	0,038001	0,033017	0,8688	0,04352

hsa-miR-17-5p	0,035087	0,033649	0,959	0,21917

hsa-miR-30b-5p	0,060524	0,035303	0,5833	0,00026

hsa-miR-130a-3p	0,091567	0,048918	0,5342	0,00279

hsa-miR-30e-5p	0,38221	0,05628	0,1472 ˅	0,00114

hsa-miR-16-5p	1,916412	0,094838	0,008 ˅	0,0456

hsa-miR-30a-5p	0,05877	0,04679	0,7962	0,11647

˅, down-regulation (blue).

Fold-change values less than 0.5 is indicated in blue.

The expressions of hsa-miR-21-5p, hsa-miR-142-3p, hsa-miR-424-5p, hsa-miR-223-3p, hsa-miR-26a-5p, hsa-miR-106b-5p, hsa-miR-126-5p, hsa-miR-181a-5p, hsa-miR-222-3p, hsa-miR-22-3p, hsa-miR-24-3p, hsa-miR-17-3p, hsa-miR-30b-5p, hsa-miR-130a-3p, hsa-miR-30e-5p, and hsa-miR-16-5p were found to be significantly down-regulated in the psoriasis group when compared with control group (*p* < 0.05; Table 3). Fold changes in the patient group compared to the control group are also presented in Table 2, Table 3.

In the patient group, no significant relationship was observed between mi-RNA expressions and PASI score, DLQI score, and nail involvement.

## Discussion

Psoriasis is a chronic inflammatory skin disease in which epidermal keratinocytes, immune cells, and inflammatory mediators are involved in the pathogenesis. In recent years, it was shown that mi-RNAs regulate differentiation, proliferation, and cytokine responses of keratinocytes, survival, and activation of T-cells, as well as the association between immunocytes and keratinocytes. Although different expression profiles of mi-RNAs have been reported in psoriasis patients, mi-RNAs have not been identified as a biomarker for diagnosis, disease activity, and disease severity in clinical practice.^17^ Although most of patients included in the present study presented milder disease, up- and down-regulations in the mi-RNA expressions in serum samples of psoriasis patients were observed; the present findings were compatible with the existing literature data on serum and skin samples of psoriasis patients.^18^, ^19^, ^20^ However, in the present study, these mi-RNA expressions were not significantly associated with the PASI score, DLQI score, and nail involvement.

Many mi-RNAs involving miR-203 and miR-125b have been shown to play key roles in inflammatory response, immune dysfunction, and hyperproliferative diseases, including psoriasis. It has been suggested that mi-RNAs detected from blood samples may be biomarkers in diagnosis, prognosis, as well as treatment response.^21^ In the present study, while miR-146a and miR125b were found to be up- and down-regulated in psoriasis patients, respectively, the association was not statistically significant.

The expression of diverse mi-RNAs was shown to be related to psoriasis activity or severity in several studies. In the study by Hou RX et al., miR-155-5p expression was found to increase in dermal mesenchymal stem cells in psoriasis patients.^18^ Guo et al. have observed that miR-369-3p expression was increased in serum and skin of psoriasis patients and associated with disease activity.^19^ In another study, miR-1266-5p expression was reported as increased in the serum of psoriasis patients and associated with disease activity.^20^ Similarly to these studies, in the present study miR-155-5p, miR-369-3P, and miR-1266-5p expressions were significantly up-regulated in psoriasis patients when compared with controls. However, no statistically significant association was observed with miRNAs and disease severity. This result may be due to the fact that the majority of the present patients had mild or moderate psoriasis.

The effect of diverse systemic treatments on mi-RNA expressions and the role of mi-RNAs in the pathogenesis of the disease have been investigated. Raaby et al. detected no changes in mi-RNA expression four days after adalimumab treatment, suggesting that the cytokine expression profile is not facilitated by early changes in mi-RNA expression.^22^ In contrast to the present study, miR-142-3p levels were increased in psoriatic patients and decreased with etanercept treatment in the study by Pivarcsi et al.^23^ Ichıhara et al. have found decreased miR-424 expression in the tissues of psoriasis patients, and observed that decreased miR-424 associated increased MEK1 and cyclin E1 levels may induce keratinocyte proliferation in the epidermis.^24^ Similarly, in the present study miR-424 expression was down-regulated in psoriasis patients when compared with controls.

Studies on miRNAs and other chronic diseases may also be useful in understanding the pathogenesis and effect mechanism of systemic treatments in psoriasis. In a study conducted on patients with rheumatoid arthritis, Xiang et al. observed that increased miR-498 expression suppressed Th17 cell differentiation by targeting STAT3.^25^ In another study by Wang et al., miR-223 was shown to cause inflammatory bowel disease through the IL23 pathway.^26^ It is known that the IL23/17 pathway plays a key role in psoriasis pathogenesis, and treatments against this pathway, such as anti-IL17 (secukinumab), are effective in the treatment of psoriasis.^27^, ^28^ Consequently, overexpression of miR-498 may be effective in the treatment of psoriasis through the IL17 pathway. Although the authors did not investigate mi-RNA expressions before and after the treatments, a significantly up-regulated expression of miR-498 was observed in psoriasis patients. In another study, An et al. demonstrated that the upregulation of miR-205 inhibits the development of keloid by suppressing vascular endothelial growth factor (VEGF) production.^29^ Chamorro-Jorganes et al. have reported that VEGF contributes to angiogenesis in endothelial cells by inducing miR-17.^30^ Since VEGF has been known to be involved in psoriasis pathogenesis, these reports support the hypothesis that miR-205 and miR-17 expressions may affect psoriasis development via VEGF.^31^ In the present study, while miR-205-5p expression was significantly up-regulated, mir-17-3p expression was significantly down-regulated in psoriasis patients when compared with controls.

Various mi-RNA types have been observed to be involved in the pathogenesis of diverse psoriasis types. While the IL23/Th17 pathway is responsible for the development of psoriasis vulgaris, IL-1, IL-36, and IL-6 are the cytokines known to be involved in the development of pustular psoriasis.^32^ Chyi-Ying et al. reported that miR-26 reduces IL-6, which is activated by TNF-α.^33^ MiR-26 may be involved in the development of IL-6 mediated psoriasis. Drugs that suppress IL-6 release, such as tocilizumab, are used in the treatment of rheumatoid arthritis and juvenile rheumatoid arthritis, and are among the possible future treatment options of pustular psoriasis.^32^

Signal transducer and transcription activation (STAT3) has recently emerged as a key player in the pathogenesis of psoriasis and psoriasis-like inflammatory conditions. It has been reported that STAT3 hyperactivity mediates the signaling of most cytokines, including interleukin IL-23/IL-17, and is observed in almost every cell type involved in disease onset.^34^ Maimaiti et al. have reported that miR-106b modulates angiogenesis in endothelial cells by affecting STAT3 expression.^35^ In another study, STAT3 was shown to inhibit miR-22 expression in patients with cutaneous T-cell lymphoma.^36^ Thus, miR-22 may be inhibited by STAT3 mediated pathogenesis in psoriasis. In the present study, miR-106b-5p and miR-22 expressions were found to be significantly up- and down-regulated in psoriasis patients, respectively, supporting these data.

The present study has limitations: only patients with mild to moderate psoriasis were included, and most of them were undergoing topical treatment only; therefore, about it was not possible to assess the changes in miRNA expressions before and after systemic treatments. Additionally, the present patients had plaque-type psoriasis and thus, mi-RNA expressions was not assessed in other types of psoriasis.

## Conclusion

In conclusion, while the present study included patients with mild to moderate psoriasis and assessed miRNAs from the blood samples rather than skin lesions, a certain panel of mi-RNAs were up- or down-regulated in the psoriasis patients, compatible with the existing literature data. Therefore, the present results once again support the role of mi-RNAs in the psoriasis pathogenesis. However, no association was observed between mi-RNA expressions and PASI score, DLQI score, and nail involvement in patients. Further studies should be conducted to better understand whether mi-RNAs can be used as a marker for psoriasis prognosis, which mi-RNA is more specific for which type of psoriasis, and whether miRNAs can be used in the treatment of psoriasis in the future.

## Financial support

None declared.

## Authors’ contributions

Emine Tugba Alatas: Study design and planning; Drafting of the manuscript; Data collection, analysis and interpretation; Effective participation in research orientation; Intellectual participation in the propedeutics/therapeutic management of the studied cases.

Murat Kara: Statistical analysis; Study design and planning; Data collection, analysis and interpretation.

Gursoy Dogan: Approval of the final version of the manuscript; critical review of the manuscript.

Aslı Akın Belli: Approval of the final version of the manuscript; critical review of the literature; critical review of the manuscript.

## Conflicts of interest

None declared.
